# CME ACTIVITY: Clinical Characteristics and Molecular Subtyping of *Vibrio vulnificus* Illnesses, Israel

**DOI:** 10.3201/eid1412.080498

**Published:** 2008-12

Medscape, LLC is pleased to provide online continuing medical education (CME) for this journal article, allowing clinicians the opportunity to earn CME credit. Medscape, LLC is accredited by the Accreditation Council for Continuing Medical Education (ACCME) to provide CME for physicians. Medscape, LLC designates this educational activity for a maximum of 0.75 *AMA PRA Category 1 Credits*™. Physicians should only claim credit commensurate with the extent of their participation in the activity. All other clinicians completing this activity will be issued a certificate of participation. To participate in this journal CME activity: (1) review the learning objectives and author disclosures; (2) study the education content; (3) take the post-test and/or complete the evaluation at **http://www.medscape.com/cme/eid**; (4) view/print certificate.

## Learning Objectives

Upon completion of this activity, participants will be able to:

- List the predisposing factors for infections caused by *Vibrio vulnificus* biotypes 1 and 2.
- Identify the differences between infections caused by *V. vulnificus* biotypes 1 and 2 and biotype 3.
- Describe the types of fish associated with *V. vulnificus* biotype 3 infection.
- Describe the mortality associated with *V. vulnificus* biotype 3 infection.
- List the predictors of mortality in *V. vulnificus* biotype 3 infection.

## Editor

**Beverly Merritt**, Technical Writer-Editor, *Emerging Infectious Diseases*. *Disclosure: Beverly Merritt has disclosed no relevant financial relationships.*

## CME AUTHOR

**Désirée Lie, MD, MSEd**, Clinical Professor, Family Medicine, University of California, Orange; Director, Division of Faculty Development, UCI Medical Center, Orange, California. *Disclosure: Désirée Lie, MD, MSEd, has disclosed no relevant financial relationships.*

## AUTHORS

Disclosures: **Ronit Zaidenstein, MD; Chantal Sadik, MD; Larisa Lerner, MD; Lea Valinsky, PhD; June Kopelowitz, PhD; Ruth Yishai, PhD; Vered Agmon, PhD; Michele Parsons, MS; Cheryl Bopp, MS;** and **Miriam Weinberger, MD,** have disclosed no relevant financial relationships.

## Earning CME Credit

To obtain credit, you should first read the journal article. After reading the article, you should be able to answer the following, related, multiple-choice questions. To complete the questions and earn continuing medical education (CME) credit, please go to **http://www.medscape.com/cme/eid**. Credit cannot be obtained for tests completed on paper, although you may use the worksheet below to keep a record of your answers. You must be a registered user on Medscape.com. If you are not registered on Medscape.com, please click on the New Users: Free Registration link on the left hand side of the website to register. Only one answer is correct for each question. Once you successfully answer all post-test questions you will be able to view and/or print your certificate. For questions regarding the content of this activity, contact the accredited provider, CME@medscape.net. For technical assistance, contact CME@webmd.net. American Medical Association’s Physician’s Recognition Award (AMA PRA) credits are accepted in the US as evidence of participation in CME activities. For further information on this award, please refer to http://www.ama-assn.org/ama/pub/category/2922.html. The AMA has determined that physicians not licensed in the US who participate in this CME activity are eligible for *AMA PRA Category 1 Credits*™. Through agreements that the AMA has made with agencies in some countries, AMA PRA credit is acceptable as evidence of participation in CME activities. If you are not licensed in the US and want to obtain an AMA PRA CME credit, please complete the questions online, print the certificate and present it to your national medical association.

### Article Title: Clinical Characteristics and Molecular Subtyping of *Vibrio vulnificus* Illnesses, Israel

## CME Questions

1. Which of the following is least likely to be a predisposing factor for infections caused by *Vibrio vulnificus* biotypes 1 and 2?
  A. Anemia
  B. Organ transplantation
  C. HIV infection
  D. Chronic liver disease
2. Illness caused by *V. vulnificus* biotype 3 is best distinguished from that caused by biotypes 1 and 2 by which of the following?
  A. Method of transmission
  B. Genetic features
  C. Geographic distribution
  D. All of the above
3. Which of the following is the primary fish involved in *V. vulnificus* biotype 3 infection?
  A. Gray mullet
  B. Tilapia
  C. Salmon
  D. Sea bass
4. Which of the following best describes the mortality associated with *V. vulnificus* biotype 3 infection as reported in Israel?
  A. 1.0%
  B. 2.3%
  C. 5.2%
  D. 7.5%
5. Which of the following is least likely to be an independent predictor of mortality for *V. vulnificus* biotype 3 infection?
  A. Bacteremia
  B. Ischemic heart disease
  C. Sex
  D. Altered immune status

### Activity Evaluation

**Table:** 

**1. The activity supported the learning objectives.**				
Strongly Disagree				Strongly Agree
1	2	3	4	5
**2. The material was organized clearly for learning to occur.**				
Strongly Disagree				Strongly Agree
1	2	3	4	5
**3. The content learned from this activity will impact my practice.**				
Strongly Disagree				Strongly Agree
1	2	3	4	5
**4. The activity was presented objectively and free of commercial bias.**				
Strongly Disagree				Strongly Agree
1	2	3	4	5

